# Varicella-zoster virus-associated central nervous system infection in immunocompromised vs. immunocompetent herpes zoster patients: a comparative study

**DOI:** 10.3389/fimmu.2026.1795710

**Published:** 2026-04-02

**Authors:** Jun Wang, Yanrong Yuan, Yan Zhang, Huili Liu, Jing Han, Yongxing Yan

**Affiliations:** Department of Neurology, Hangzhou Third People’s Hospital, Zhejiang, Hangzhou, China

**Keywords:** encephalitis, herpes zoster, immunity, meningitis, varicella-zoster virus

## Abstract

**Objectives:**

Varicella-zoster virus (VZV) is a significant pathogen of viral central nervous system (CNS) infections. There are many studies on VZV-associated CNS infections, but there is currently a lack of large-sample comparative studies on VZV-associated CNS infections in immunocompetent and immunocompromised herpes zoster (HZ) patients. In this study, we compared the clinical characteristics and prognosis of VZV-associated CNS infections in immunocompetent and immunocompromised HZ patients.

**Methods:**

This retrospective study, conducted at a tertiary hospital specializing in dermatology in East China, compares clinical characteristics, treatment, and prognosis at discharge in 117 immunocompetent and 49 immunocompromised HZ patients with VZV-associated CNS infection.

**Results:**

Compared with the immunocompetent group, the incidence of encephalitis in immunocompromised HZ patients with VZV-associated CNS infections was significantly higher (P<0.05), and the prognosis at discharge was worse (P<0.01). Blood white blood cell (WBC), red blood cell (RBC), blood platelet counts (BPC), hemoglobin (Hb), total protein, prealbumin, and albumin contents decreased (all P<0.05), while adenosine deaminase (ADA) levels in cerebrospinal fluid (CSF) and blood C-reactive protein-to-albumin ratio (CAR), systemic immune-inflammation index (SII), C-reactive protein (CRP), and β2 microglobulin levels were higher (all P<0.05). Further analysis revealed that the time between neurological symptoms and antiviral therapy and the occurrence of encephalitis were independent risk factors for poor prognosis at discharge in immunocompetent HZ patients with CNS infections, while the independent risk factors for poor prognosis in the immunocompromised group were age and the time between neurological symptoms and antiviral therapy.

**Conclusions:**

Immunocompromised HZ patients have a higher incidence of encephalitis and worse prognosis. Regardless of immunocompetent or immunocompromised HZ patients with concurrent CNS infections, the time between neurological symptoms and antiviral therapy is an independent risk factor for prognosis at discharge, highlighting the importance of early intervention and treatment.

## Introduction

Herpes zoster(HZ) is a skin infectious disease caused by reactivation of the varicella-zoster virus (VZV), which is latent in the host (1). VZV, also known as human herpes virus 3, has a high affinity for skin and nerve tissue (2). After the first infection, the virus can remain dormant in the ganglia for a long time. Dormant VZV can reactivate and replicate in large quantities. The reactivated VZV can reach the nerve innervation area along the nerve axon, causing shingles symptoms such as erythema, blisters, and neuralgia on one side of the dermatome. Immune dysfunction is a significant risk factor for the occurrence of HZ in the host (3–6).

The majority of HZ patients can be completely cured after standardized treatment, but some patients still develop peripheral nervous system involvement, such as postherpetic neuralgia (PHN), herpes zoster ophthalmopathy, and cranial nerve paralysis. In some patients, VZV can involve the central nervous system (CNS), resulting in complications such as meningitis, encephalitis, myelitis, and cerebrovascular disease (7–9). Among them, meningitis and encephalitis are the main complications of VZV-associated CNS infections (10, 11). Tobias Tyrberg et al. (10) conducted a nationwide Swedish retrospective case-control study, which included 1488 patients with VZV-associated CNS infections. Among them, meningitis was the most frequent (45%), followed by encephalitis (38%). Elderly individuals have an increased risk of developing CNS infections and are more susceptible to encephalitis. HIV, hematological cancer, and treatment with specific immunosuppressants or high-dose glucocorticoids were significant risk factors for VZV-associated CNS infection. Laura Krogh Herlin et al. (12) conducted a retrospective analysis of clinical data from 92 VZV encephalitis patients nationwide in Denmark, which revealed that age and immunosuppression are high-risk factors for VZV encephalitis. In 2025, Ci XJ et al. (13) retrospectively analyzed the clinical characteristics of 108 immunocompetent patients diagnosed with VZV CNS infection and found that both VZV meningitis and encephalitis in immunocompetent individuals typically present with mild clinical symptoms and have a good prognosis.

Although there are increasing studies of CNS infections caused by VZV reactivation in clinical practice, few studies have examined the differences between immunocompromised and immunocompetent HZ patients with CNS infection, although CNS infections are more frequently reported in immunocompromised patients (14–16). Therefore, this study systematically compared the clinical characteristics, treatment, and prognosis of VZV-associated CNS infections in immunocompetent and immunocompromised patients hospitalized in our center from January 2020 to December 2025, in order to provide a reference for clinicians in the diagnosis and treatment of VZV-associated CNS infection.

## Materials and methods

### Methods

A retrospective comparative study was conducted at the Department of Neurology, Hangzhou Third People’s Hospital, Zhejiang, China, a tertiary hospital specializing in dermatology in East China. Clinical data were collected from the electronic medical records system, including the diagnosis, treatment, and follow-up of patients with VZV-associated meningitis/encephalitis hospitalized in our center from January 2020 to December 2025. The collected data included sex, age, location of shingles, course of disease, comorbidities, clinical symptoms, CSF, cranial CT/MRI, electroencephalogram, the time between shingles and neurological symptoms, the time between neurological symptoms and antiviral therapy, hospitalization days, and prognosis at discharge. This study was approved by the Ethics Committee of Hangzhou Third People’s Hospital (No. 2021KA013).

Patients with VZV-associated meningitis/encephalitis were included according to our previous criteria (17, 18). (The diagnosis of HZ met the criteria of the European consensus-based guideline (19), and the diagnosis of VZV encephalitis met the consensus statement of the International Encephalitis Consortium (20)). In brief, for shingles within 4 weeks, patients with symptoms/signs of CNS infection, CSF white blood cell counts >5×10^6^/L or protein levels > 45mg/dL or VZV DNA (+), and metagenomic next-generation sequencing (mNGS) detection of pathogenic microorganisms in CSF excluding bacterial, fungal, and other microbial infections were included. All included patients had no history of VZV vaccination and did not receive antiviral treatment before admission.

Immunocompromised individuals were defined by previous study (21) as those with impaired immune function. These included solid organ malignancies, hematological malignancies, solid organ transplantation, hematopoietic stem cell transplantation, human immunodeficiency virus (HIV) infection (CD4^+^< 400×10^6^/L), end-stage renal disease, congenital immune deficiencies, or autoimmune diseases undergoing chemotherapy or glucocorticoid treatment (taking prednisone ≥20mg/day or an equivalent dose of corticosteroids for at least one month). Individuals with shingles were defined as immunocompromised HZ patients, whereas patients without these conditions were defined as immunocompetent HZ patients.

Prognosis was assessed at discharge and categorized according to the Glasgow Outcome Scale (GOS), which ranges from 1 to 5 points. Prognosis at discharge was divided into two categories: Good: GOS ≥ 4 points and Poor: GOS ≤ 3 points. Neurologists in our research team assessed the prognosis of patients.

### Statistical analysis

Data processing and statistical analysis were performed using SPSS 26.0 software. The Kolmogorov-Smirnov test was used to assess the normality of each group. Measurement data conforming to a normal distribution were expressed as x ± sd. The t-test was used for comparing the means of two samples. For data with non-normal distribution, the median (interquartile range) (M [P25, P75]) was used for description, and the Mann-Whitney U test was used for comparing between two groups. Counting data were expressed as frequency and percentage, and the chi-square test or Fisher’s exact test was used for group comparisons. Factors with statistical significance in univariate analysis were selected for multivariate binary logistic regression analysis. P<0.05 was considered statistically significant.

## Results

### Baseline characteristics of VZV-associated CNS infection in immunocompetent and immunocompromised HZ patients

According to the criteria for distinguishing immune function and the diagnostic criteria of VZV-associated CNS infection, 117 patients were included in the immunocompetent VZV-associated CNS infection group, including 72 males and 45 females, with an average age of 51.7 ± 15.8 years. There were 49 patients in the immunocompromised VZV-associated CNS infection group, including 27 males and 22 females, with an average age of 65.4 ± 11.3 years. The baseline characteristics of both groups are shown in **Table 1**. Patients tended to be younger (51.7 ± 15.8 vs 65.4 ± 11.3 years; standardized mean differences (SMD)=0.97) in the immunocompetent HZ group, which also had a higher proportion of males (61.5% vs 55.1%; SMD = 0.13) and a lower proportion of hypertension comorbidity (24.8% vs 38.8%; SMD = 0.21). The head was the leading location of shingles of VZV-associated CNS infection in both immunocompetent and immunocompromised HZ patients (72.6% vs 63.3%, respectively; SMD = 0.11). All included patients underwent mNGS testing for pathogenic microorganisms in CSF. The results showed that 69 out of 117 immunocompetent patients (69/117, 59.0%) and 34 out of 49 immunocompromised patients (34/49, 69.4%) had VZV DNA (+) in CSF.

**Table 1:** Baseline characteristics of immunocompetent and immunocompromised HZ patients with VZV-associated CNS infection.

Characteristics	Immunocompetent (n=117)	Immunocompromised (n=49)	Absolute SMD
Age(x ± sd)	51.7 ± 15.8	65.4 ± 11.3	0.97
Sex.(n,%)			
Male	72(61.5%)	27(55.1%)	0.13
Female	45(38.5%)	22(44.9%)	
Comorbidities(n,%)			
Hypertension	29(24.8%)	19(38.8%)	0.21
Type 2 diabetes	14(12.0%)	9(18.4%)	0.19
Coronary heart disease	4(3.4%)	5(10.2%)	0.22
Stroke	4(3.4%)	5(10.2%)	0.22
Location of shingles(n,%)			
Head	85(72.6%)	31(63.3%)	0.11
Neck	15(12.8%)	9(18.4%)	0.06
Chest and Back	14(12.0%)	10(20.4%)	0.14
Waist and Abdomen	1(0.9%)	1(2.0%)	0.11
Upper limb	2(1.7%)	0	0.16
Lower limbs	2(1.7%)	1(2.0%)	0.03

SMD, standardized mean differences.

### Clinical manifestations

**Figure 1:**
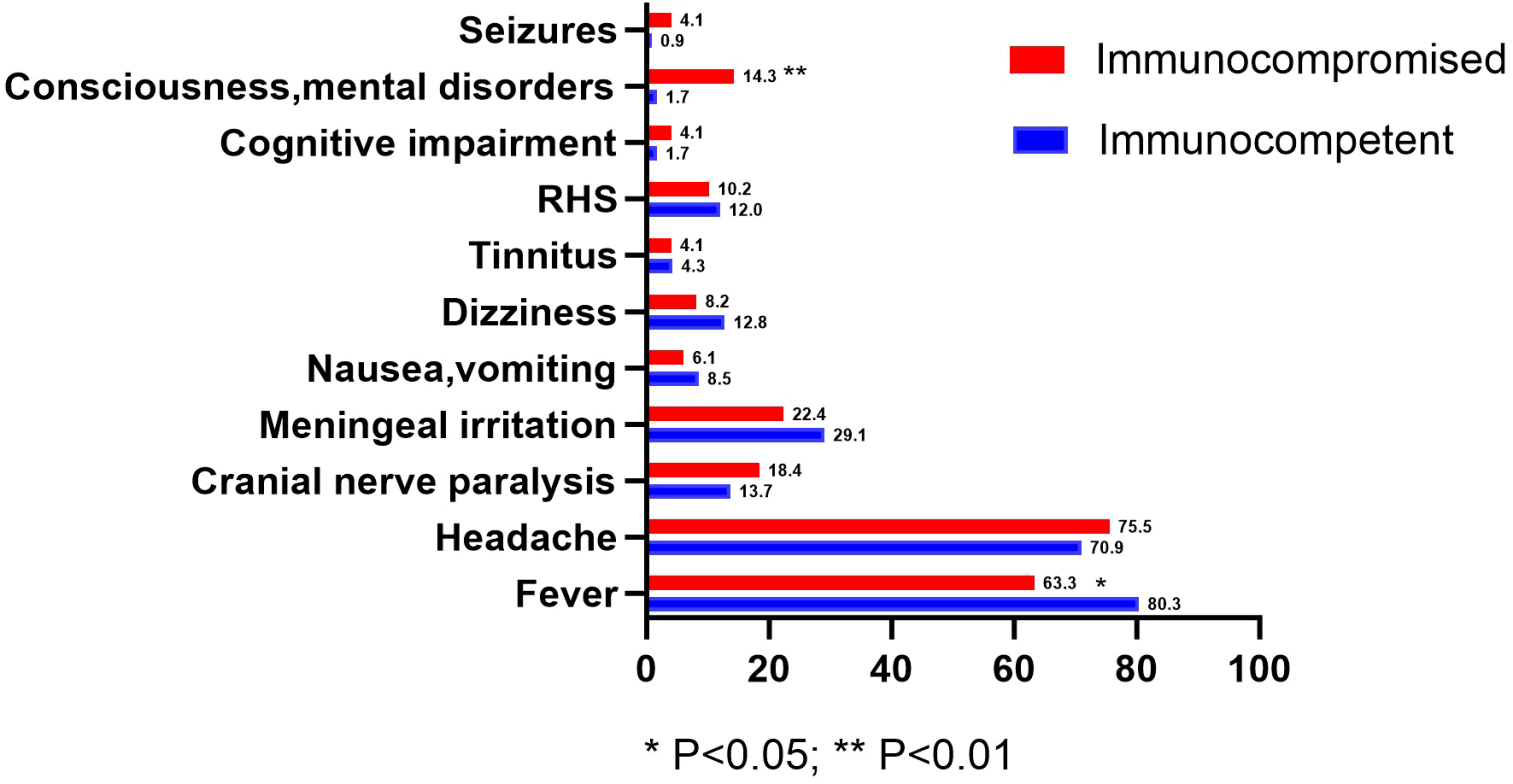
Clinical manifestations of patients in the two groups (%).

The most common clinical manifestations among the 117 immunocompetent patients were fever (80.3%), headache (70.9%), meningeal irritation (29.1%), cranial nerve paralysis (13.7%), and dizziness (12.8%). In contrast, the common clinical manifestations in 49 immunocompromised patients were headache, fever, meningeal irritation, cranial nerve paralysis, and consciousness/mental disorders, accounting for 75.5%, 63.3%, 22.4%, 18.4%, and 14.3%, respectively. The proportion of fever in immunocompromised patients was lower than that in immunocompetent patients (P<0.05), while the proportion of consciousness/mental disorders was significantly higher (P<0.01) (**Figure 1**).

### Blood and CSF testing in two groups

Compared with the immunocompetent group, immunocompromised patients had decreased levels of white blood cell (WBC), red blood cell (RBC), hemoglobin (Hb), blood platelet count (BPC), total protein, prealbumin, and albumin (all P<0.05), but increased levels of C-reactive protein-to-albumin ratio (CAR), systemic immune-inflammation index (SII), C-reactive protein (CRP), and β2-microglobulin (all P<0.05). CSF adenosine deaminase (ADA) levels were significantly elevated in immunocompromised patients (p<0.05) (**Table 2**).

**Table 2:** Comparison of blood and CSF tests of immunocompromised and immunocompetent HZ patients with VZV-associated CNS infection.

Characteristics	Immunocompetent (n=117)	Immunocompromised (n=49)	t/z	p
Blood				
WBC(×10^9^/L)	7.2 ± 2.8	6.2 ± 2.3	2.127^a^	0.0350
RBC(×10^12^/L)	4.6 ± 0.6	4.2 ± 0.6	3.409^a^	0.0008
Hb (g/L)	137.6 ± 22.1	128.0 ± 21.2	2.515^a^	0.0129
BPC(×10^9^/L)	210.7 ± 60.6	183.0 ± 63.1	2.577^a^	0.0109
PLR	148.3 ± 69.0	171.5 ± 101.6	1.418^a^	0.1581
NLR	3.6 ± 2.5	4.1 ± 2.6	1.146^a^	0.2535
CAR	0.09(0.01, 0.32)	0.21(0.03, 0.45)	2.829^b^	0.0053
SII	648.6 ± 443.4	852.2 ± 568.3	2.175^a^	0.0312
CRP (mg/L)	3.0(0.5, 12.1)	7.9(1.6, 16.3)	2.388^b^	0.0181
Total Protein(g/L)	63.5 ± 5.0	61.4 ± 5.8	2.293^a^	0.0232
Prealbumin(mg/L)	246.5 ± 66.2	218.7 ± 62.6	2.436^a^	0.016
Albumin(g/L)	37.0 ± 3.2	34.7 ± 3.7	3.844^a^	0.0002
Globulin(g/L)	26.7 ± 3.5	26.7 ± 4.3	0.0945^a^	0.9249
β2-microglobulin(mg/L)	1.7 ± 0.8	2.7 ± 2.0	3.614^a^	0.0004
CD4+(×10^6^/L)	561.2 ± 334.9	554.6 ± 364.9	0.0941^a^	0.9250
CD8+(×10^6^/L)	348.5 ± 207.7	350.3 ± 165.3	0.0449^a^	0.9643
CD4+/CD8+	1.8 ± 0.8	1.8 ± 1.1	0.2450^a^	0.8086
CSF				
Leukocyte count(×10^6^/L)	25.0(4.3, 81.5)	10.0(3.0, 65.5)	0.1768^b^	0.8599
Protein (mg/dl)	73.1 ± 38.8	68.4 ± 28.2	0.7738^a^	0.4402
Chlorine (mmol/L)	123.6 ± 4.6	124.1 ± 5.7	0.6760^a^	0.5000
LDH (U/L)	29.6 ± 14.0	29.2 ± 12.2	0.1985^a^	0.8429
Glucose (mmol/L)	3.9 ± 1.0	3.8 ± 1.0	0.6164^a^	0.5385
ADA (U/L)	1.2(0.6,2.0)	1.6(1.0,3.5)	2.6840^b^	0.0080

WBC, white blood cell; RBC, red blood cell; Hb, hemoglobin; BPC, blood platelet count; PLR, Platelet-to-Lymphocyte Ratio; NLR, Neutrophil-to-Lymphocyte Ratio; CAR, C-Reactive Protein-to-Albumin Ratio; SII, Systemic Immune-Inflammation Index (SII =(Platelets x Neutrophils)/Lymphocytes). LDH, lactate dehydrogenase; ADA, adenosine deaminase. Note: the column “t/z” refers to the test statistic from the t-test and the z-test, used for group comparison. ^a^: t-value; ^b^: z-value.

### Antiviral therapy regimens and prognosis at discharge in two groups

All included patients received intravenous acyclovir (10mg/kg, q8h). Among them, two cases in the immunocompetent group and one case in the immunocompromised group had renal dysfunction; these three patients had prolonged medication intervals (10mg/kg, q12h). The duration of intravenous acyclovir treatment in the immunocompetent group was 7–22 days (14.9 ± 3.6), while in the immunocompromised group it was 3–23 days (14.9 ± 4.6). There were 50 patients in the immunocompetent group who simultaneously received intravenous methylprednisolone (40-80mg/d) for 3–7 days, while 20 patients in the immunocompromised group received methylprednisolone (40-80mg/d). There were no significant differences between the two groups in the duration of antiviral treatment, use of glucocorticoids, hospitalization days, the time between shingles and neurological symptoms, or the time between neurological symptoms and antiviral therapy (all P>0.05) (**Table 3**).

**Table 3:** Antiviral therapy regimens and prognosis at discharge in immunocompromised and immunocompetent HZ patients.

Characteristics	Immunocompetent (n=117)	Immunocompromised (n=49)	t/z/x^2^	p
Time between shingles and neurological symptom (days), (M[P25, P75])	4.0(1.0, 7.0)	4.0(1.0, 7.0)	0.1251^b^	0.9006
Time between neurological symptoms and antiviral therapy(days), (M[P25, P75])	3.0(1.0, 5.0)	2.0(1.0, 5.0)	0.7030^b^	0.4831
Hospitalization days(x ± sd)	16.7 ± 4.5	17.8 ± 6.4	1.302^a^	0.1946
Treatment				
Days of antiviral treatment(x ± sd)	14.9 ± 3.6	14.9 ± 4.6	0.0121^a^	0.9904
Using glucocorticoids(n)	50	20	0.0521^c^	0.8194
VZV-associated CNS infection(n)				
Meningitis	111	39	9.2581^c^	0.0023
Encephalitis	6	10		
Prognosis at discharged(n)				
Good	104	37	4.8323^c^	0.0279
Poor	13	12		

the column “t/z/χ²” refers to the test statistic from the t-test, z-test, or chi-square test, respectively, used for group comparison. ^a^: t-value; ^b^: z-value; ^c^:χ²-value.

Among the 117 immunocompetent patients with VZV-associated CNS infection, there were 111 cases of VZV meningitis and 6 cases of VZV encephalitis. A total of 104 cases had a good prognosis and 13 cases had a poor prognosis at discharge. Among the 49 immunocompromised patients, there were 39 cases of VZV meningitis and 10 cases of VZV encephalitis. A total of 37 cases had a good prognosis and 12 cases had a poor prognosis at discharge. The proportion of VZV encephalitis in immunocompromised patients was significantly higher than in immunocompetent patients (P<0.01), and the prognosis was worse at discharge (P<0.05, **Table 3**).

### Factors of prognosis in 117 immunocompetent HZ patients with VZV-associated CNS infection

Based on the prognosis criteria for patients at discharge, there were 104 patients (104/117, 88.9%) in the good prognosis group, including 62 males and 42 females, with an average age of 51.2 ± 16.0 years. Among them, 101 cases (97.1%) were VZV meningitis and 3 cases (2.9%) were VZV encephalitis. There were 13 patients (13/117, 11.1%) in the poor prognosis group, including 10 males and 3 females, with an average age of 55.8 ± 13.8 years. Among them, 10 cases (76.9%) were VZV meningitis and 3 cases (23.1%) were VZV encephalitis. The incidence of encephalitis was significantly higher in the poor prognosis group (P<0.01) (**Table 4**).

**Table 4:** Comparison of the characteristics of different prognosis at discharge in 117 immunocompetent patients with VZV-associated CNS infection.

Characteristics	Good (n=104)	Poor (n=13)	t/z/x^2^	p
Age(x ± sd)	51.2 ± 16.0	55.8 ± 13.8	1.007^a^	0.3159
Sex.(n)				
Male	62	10	1.4625^c^	0.2265
Female	42	3		
VZV-associated CNS infection(n)				
Meningitis	101	10	9.6841^c^	0.0019
Encephalitis	3	3		
Time between shingles and neurological symptoms(days), (M[P25, P75])	4.0(1.3,7.0)	3.0(1.0,7.0)	2.158^b^	0.0330
Time between neurological symptoms and antiviral therapy(days), (M[P25, P75])	2.0(1.0,4.8)	5.0(3.0,19.0)	3.526^b^	0.0006
Hospitalization days(x ± sd)	16.4 ± 4.3	18.9 ± 5.1	1.1945^a^	0.0542
Treatment				
Days of antiviral treatment(x ± sd)	14.7 ± 3.5	16.2 ± 3.6	1.426^a^	0.1567
Using glucocorticoids(n)	43	7	0.7378^c^	0.3904
Blood				
WBC(×10^9^/L)	7.2 ± 2.8	6.8 ± 1.9	0.5761^a^	0.5660
RBC(×10^12^/L)	4.6 ± 0.6	4.5 ± 0.7	0.5088^a^	0.6119
Hb(g/L)	137.9 ± 22.6	135.4 ± 17.2	0.3628^a^	0.7175
BPC(×10^9^/L)	213.3 ± 61.4	188.7 ± 50.2	1.3360^a^	0.1843
PLR	169.6 ± 102.8	187.5 ± 93.2	0.5767^a^	0.5653
NLR	4.0 ± 2.6	4.9 ± 2.6	1.082^a^	0.2816
CAR	0.09(0.01,0.32)	0.03(0.01,0.64)	0.6357^b^	0.5264
SII	846.3 ± 579.1	902.0 ± 486.2	0.3195^a^	0.7499
CRP (mg/L)	3.0(0.5,11.3)	1.4(0.5,26.4)	2.358^b^	0.0201
Total Protein(g/L)	63.6 ± 4.8	62.3 ± 6.2	0.8915^a^	0.3746
Prealbumin(mg/L)	243.9 ± 64.6	268.3 ± 77.8	1.207^a^	0.2299
Albumin(g/L)	37.1 ± 3.0	35.3 ± 4.4	1.6610^a^	0.0996
Globulin(g/L)	26.5 ± 3.1	28.0 ± 5.7	1.4290^a^	0.1558
β2-microglobulin(mg/L)	1.7 ± 0.5	2.6 ± 2.5	2.747^a^	0.0074
CD4+(×10^6^/L)	587.5 ± 338.2	301.3 ± 134.1	2.508^a^	0.0138
CD8+(×10^6^/L)	357.8 ± 211.7	256.3 ± 139.7	1.404^a^	0.1635
CD4+/CD8+	1.8 ± 0.9	1.4 ± 0.7	1.041^a^	0.1266
CSF				
Leukocyte counts(×10^6^/L)	30.0(4.0,85.0)	8.0(4.0,26.5)	0.9647^b^	0.3367
Protein (mg/dl)	74.7 ± 39.7	60.7 ± 29.6	1.229^a^	0.2218
Chlorine (mmol/L)	123.5 ± 4.7	124.1 ± 4.5	0.4168^a^	0.6776
LDH (U/L)	29.9 ± 13.9	27.3 ± 15.9	0.6284^a^	0.5310
Glucose (mmol/L)	3.9 ± 1.0	4.1 ± 0.8	0.7622^a^	0.4475
ADA (U/L)	1.1(0.5,2.0)	1.2(0.95,3.0)	1.472^b^	0.1440

the column “t/z/χ²” refers to the test statistic from the t-test, z-test, or chi-square test, respectively, used for group comparison. ^a^: t-value; ^b^: z-value; ^c^:χ²-value.

Patients in the poor prognosis group had a shorter time between shingles and neurological symptoms (P<0.05) and a longer time between neurological symptoms and antiviral therapy (P<0.01). However, there were no significant differences in the duration of antiviral treatment, use of glucocorticoids, or hospitalization days between the two groups (P>0.05). In the poor prognosis group, CRP and β2-microglobulin levels were elevated (P<0.05, P<0.01), while blood CD4+ levels were decreased (P<0.05) (**Table 4**).

### Independent risk factors for prognosis at discharge in immunocompetent HZ patients with CNS infections

Using prognosis at discharge (Good: GOS≥4 points; Poor: GOS ≤ 3 points) as the dependent variable, a multivariate binary logistic regression analysis was conducted with factors that were statistically significant (P<0.05) in the univariate analysis (occurrence of encephalitis, time between shingles and neurological symptoms, time between neurological symptoms and antiviral therapy, blood CRP, β2 microglobulin, and CD4+ levels) as independent variables. After adjusting for confounding factors such as age, sex, and treatment, it was found that the occurrence of encephalitis and the time between neurological symptoms and antiviral therapy were independent risk factors for prognosis at discharge in immunocompetent HZ patients with CNS infections (all P<0.05), while CD4+ levels were a protective factor (P<0.01). Factors such as the time between shingles and neurological symptoms, blood CRP, and β2 microglobulin levels were not independent risk factors (P>0.05) (**Table 5**).

**Table 5:** Binary logistic regression analysis of the independent risk factors for prognosis at discharge in immunocompetent HZ patients with CNS infections.

Variable	β	Wald	P	Exp.	95%CI
Occurrence of encephalitis	0.359	7.438	0.006	1.432	1.106-1.854
Time between shingles and neurological symptoms(days)	0.048	0.414	0.520	1.049	0.907-1.214
Time between neurological symptoms and antiviral therapy(days)	0.094	4.247	0.039	1.098	1.005-1.201
CRP (mg/L)	0.024	0.540	0.463	1.024	0.961-1.092
β2-microglobulin (mg/L)	0.042	0.019	0.891	1.043	0.572-1.902
CD4+ (×10^6^/L)	-0.008	5.250	0.022	0.992	0.985-0.999

### Factors of prognosis in 49 immunocompromised HZ patients with VZV-associated CNS infection

Based on the prognosis criteria for patients at discharge, 37 out of 49 patients (37/49, 75.5%) had a good prognosis. Among them, there were 18 males and 19 females, with an average age of 63.3 ± 11.4 years. This group included 32 cases of VZV meningitis (86.5%) and 5 cases of VZV encephalitis (13.5%). The poor prognosis group comprised 12 patients (12/49, 24.5%), including 9 males and 3 females, with an average age of 72.1 ± 8.3 years. This group included 7 cases of VZV meningitis (58.3%) and 5 cases of VZV encephalitis (41.7%). There were significant differences in age and the incidence of VZV encephalitis between the two groups (P<0.05). Patients in the poor prognosis group were older and had a significantly higher incidence of encephalitis (P<0.05) (**Table 6**).

**Table 6:** Comparison of the characteristics of different prognosis at discharge in 49 immunocompromised patients with VZV-associated CNS infection.

Characteristics	Good (n=37)	Poor (n=12)	t/z/x^2^	p
Age(x ± sd)	63.3 ± 11.4	72.1 ± 8.3	2.467^a^	0.0173
Sex.(n)				
Male	18	9	2.5433^c^	0.1108
Female	19	3		
VZV-associated CNS infection(n)				
Meningitis	32	7	4.4215^c^	0.0355
Encephalitis	5	5		
Time between shingles and neurological symptoms(days), (M[P25, P75])	4.0(1.0,7.0)	4.0(1.0,6.8)	0.4614^b^	0.6466
Time between neurological symptoms and antiviral therapy(days), (M[P25, P75])	2.0(1.0,4.0)	6.0(2.0,10.8)	3.317^b^	0.0018
Hospitalization days(x ± sd)	17.7 ± 5.0	18.2 ± 9.9	0.2153^a^	0.8305
Treatment				
Days of antiviral treatment(x ± sd)	15.3 ± 4.0	13.6 ± 6.1	1.147^a^	0.2572
Using glucocorticoids(n)	13	7	2.0186^c^	0.1554
Blood				
WBC(×10^9^/L)	6.4 ± 2.4	5.8 ± 1.5	0.7764^a^	0.4416
RBC(×10^12^/L)	4.2 ± 0.6	4.3 ± 0.5	0.5034^a^	0.6172
Hb(g/L)	127.7 ± 21.4	129.0 ± 21.1	0.1745^a^	0.8623
BPC(×10^9^/L)	191.8 ± 66.8	155.1 ± 39.8	1.721^a^	0.0922
PLR	142.2 ± 69.9	167.7 ± 65.4	1.069^a^	0.2909
NLR	3.2 ± 1.9	5.0 ± 3.6	2.263^a^	0.0286
CAR	0.19(0.02,0.41)	0.33(0.09,1.40)	1.136^b^	0.2626
SII	623.2 ± 449.5	729.2 ± 434.1	0.6875^a^	0.4954
CRP(mg/L)	6.0(0.9,13.3)	14.0(4.0,61.2)	2.241^b^	0.0302
Total Protein(g/L)	61.4 ± 6.1	61.3 ± 5.1	0.0760^a^	0.9398
Prealbumin(mg/L)	223.8 ± 58.5	202.5 ± 74.8	0.9841^a^	0.3305
Albumin(g/L)	34.9 ± 3.8	34.2 ± 3.2	0.4941^a^	0.6240
Globulin(g/L)	26.6 ± 3.8	27.0 ± 5.7	0.2720^a^	0.7870
β2-microglobulin(mg/L)	2.3 ± 1.2	3.4 ± 3.0	1.461^a^	0.1536
CD4+(×10^6^/L)	568.5 ± 313.0	524.2 ± 477.9	0.3134^a^	0.7562
CD8+(×10^6^/L)	369.8 ± 155.6	307.4 ± 186.2	0.9897^a^	0.3302
CD4+/CD8+	1.8 ± 1.1	2.0 ± 1.1	0.4882^a^	0.6290
CSF				
Leukocyte count(×10^6^/L)	16.0(4.5,101.0)	4.0(1.3,28.3)	1.507^b^	0.1384
Protein (mg/dl)	72.3 ± 30.2	56.3 ± 16.6	1.743^a^	0.0879
Chlorine (mmol/L)	124.5 ± 5.5	123.1 ± 6.5	0.7364^a^	0.4651
LDH (U/L)	30.7 ± 13.5	24.5 ± 4.3	1.547^a^	0.1285
Glucose (mmol/L)	3.7 ± 0.9	4.3 ± 1.0	2.170^a^	0.0351
ADA (U/L)	1.2(0.9,3.5)	1.7(1.1,3.2)	0.5549^b^	0.5817

the column “t/z/χ²” refers to the test statistic from the t-test, z-test, or chi-square test, respectively, used for group comparison. ^a^: t-value; ^b^: z-value; ^c^:χ²-value.

Patients in the poor prognosis group had a longer time between neurological symptoms and antiviral therapy (P<0.01). NLR and CRP levels were also elevated (P<0.05), and CSF glucose levels were higher (P<0.05). However, there were no significant differences between the two groups in the time between shingles and neurological symptoms, duration of antiviral treatment, use of glucocorticoids, or hospitalization days (P>0.05) (**Table 6**).

### Independent risk factors for prognosis at discharge in immunocompromised HZ patients with CNS infections

Using prognosis at discharge (Good: GOS≥4 points; Poor: GOS ≤ 3 points) as the dependent variable, a multivariate binary logistic regression analysis was conducted with factors that were statistically significant (P<0.05) in the univariate analysis (age, occurrence of encephalitis, time between neurological symptoms and antiviral therapy, NLR, CRP, and CSF glucose levels) as independent variables. After adjusting for confounding factors, the results showed that age and the time between neurological symptoms and antiviral therapy were independent risk factors for prognosis at discharge in immunocompromised HZ patients with CNS infections (P<0.05), while occurrence of encephalitis, NLR, CRP, and CSF glucose levels were not independent risk factors (P>0.05) (**Table 7**).

**Table 7:** Binary logistic regression analysis of the independent risk factors for prognosis at discharge in immunocompromised HZ patients with CNS infections.

Variable	β	Wald	P	Exp.	95%CI
Age (years)	0.154	4.747	0.029	1.166	1.016-1.340
Occurrence of encephalitis	1.210	0.729	0.393	3.352	0.209-53.866
Time between neurological symptoms and antiviral therapy(days)	0.238	4.137	0.042	1.269	1.009-1.596
NLR	0.272	1.184	0.277	1.313	0.804-2.141
CRP (mg/L)	0.022	0.629	0.428	1.022	0.968-1.080
Glucose contents in CSF (mmol/L)	0.679	1.935	0.164	1.972	0.757-5.136

## Discussion

Epidemiological data indicate that the incidence of HZ is gradually increasing (22–24). Immunocompromised status is a significant risk factor for shingles in hosts (3–6). CNS infection is a severe complication in HZ patients, which can lead to severe disability or even death, significantly affecting patients’ quality of life and increasing the burden on families and society (25, 26). Recently, there have been many studies on VZV-associated CNS infections, and the clinical manifestations have become more diverse (27–30).

VZV is a common pathogen causing CNS infections and is the second most common cause of encephalitis and meningitis (31, 32). For instance, in a multi-center study from Spain (33), enterovirus meningitis was the most prevalent (76.80%), followed by VZV meningitis (10.30%). Shukla B et al. (34) also found that VZV is a common cause of aseptic meningitis, and many cases do not exhibit typical shingles manifestations (35, 36). Many studies suggest that meningitis and encephalitis are the most common types of VZV-associated CNS infections (31, 37). Despite an increasing number of clinical reports on VZV reactivation leading to CNS infections, few studies have examined the differences between immunocompromised and immunocompetent HZ patients with CNS infection. This study revealed significant differences in clinical manifestations, laboratory characteristics, and prognosis between immunocompromised and immunocompetent HZ patients who developed VZV-associated CNS infection. The most important finding was that, regardless of immune status, the time between neurological symptoms and antiviral therapy (delayed treatment) was an independent risk factor for poor prognosis at discharge. This finding provides clinical evidence for the early management of VZV-associated CNS infection and highlights the importance of early diagnosis and treatment, which is consistent with previous research results (38, 39).

HZ is a T-cell immune-related disease, and host immune dysfunction is an important risk factor for its onset (3, 4, 16). Compared to the immunocompetent group, immunocompromised HZ patients with VZV-associated CNS infection were older, with an average age difference of approximately 14 years (65.4 ± 11.3 vs 51.7 ± 15.8) in this study. This is consistent with the epidemiological characteristic that cellular immune function gradually declines with age. More importantly, patients in the immunocompromised group had a significantly higher incidence of VZV encephalitis compared to those in the immunocompetent group (20.4% vs 5.1%), and a higher proportion of poor prognosis at discharge (24.5% vs 11.1%). This is consistent with the findings of Tobias Tyrberg and Laura Krogh Herlin, which identified advanced age and immunosuppression as major risk factors for VZV-associated CNS infections, particularly encephalitis (10, 12). Meanwhile, Carlos Corral et al. (40) studied the clinical characteristics of 98 patients with VZV-associated CNS infections and also found that immunosuppression plays an important role in the severity of neurological symptoms and affects prognosis. Based on previous studies and the findings of this research, it is suggested that immunosuppression is not only a risk factor for VZV reactivation but also a significant driving factor for its progression to more severe CNS manifestations and adverse outcomes. The mechanism may be related to impaired T-cell immune function, which prevents the host from effectively controlling viral replication and diffusion within neural tissues. This makes it easier for the virus to invade the brain parenchyma, leading to more severe encephalitis rather than simple meningitis. This study found that there was no significant difference in the incidence of most clinical symptoms (such as headache, meningeal irritation, cranial nerve paralysis, nausea and vomiting, and RHS) between the two groups, reflecting the common clinical manifestations of VZV-associated CNS infections, whose specificity is relatively low. However, the proportion of consciousness/mental disorders was significantly higher, while the proportion of fever was lower in the immunocompromised group. This suggests that clinical features are mainly reflected in disease severity and atypical clinical manifestations that help distinguish between two groups. Specifically, consciousness/mental disorders are manifestations of brain parenchymal involvement, and their increased incidence is consistent with the pathological basis of higher incidence of encephalitis in immunocompromised patients, indicating more severe infection and potentially poorer prognosis in this group. On the other hand, a lower proportion of fever may reflect a blunted inflammatory response under immunosuppressive conditions, which increases the difficulty of clinical identification of severe infections and may lead to delayed diagnosis. Therefore, when evaluating patients suspected of VZV-associated CNS infection, especially those who are immunocompromised, clinicians should pay more attention to altered consciousness and realize that even without typical fever, vigilance for severe cases should be maintained.

In terms of laboratory testing, patients in the immunocompromised group exhibited distinct inflammatory and nutritional status characteristics compared to immunocompetent patients. These findings demonstrate a coexistence of an exacerbated inflammatory response and decreased nutritional reserve/hematopoietic function. A series of indicators in the peripheral blood, such as WBC, RBC, Hb, BPC, total protein, and albumin, were generally reduced in immunocompromised patients, which may reflect bone marrow suppression, malnutrition, or chronic depletion caused by underlying diseases or immunosuppressive therapy. Simultaneously, indicators representing inflammatory response (such as blood CAR, SII, CRP, β2 microglobulin, and CSF ADA levels) were significantly elevated. ADA is a key enzyme in purine metabolism, and its elevated levels reflect the activation of cellular immunity. This study found that the levels of ADA in CSF were significantly increased in immunocompromised patients, which may reveal a strong and dysregulated immune inflammatory response in the CNS. This response is consistent with the clinical characteristics of a higher incidence of encephalitis and poorer prognosis in this group, suggesting ADA as a potential biomarker for evaluating the severity of the disease. Yuan et al. (41) also found that ADA levels in CSF can predict the prognosis of patients with VZV-associated CNS infections. This coexistence of a “high inflammatory response” and ‘low nutritional reserve” likely results from the combination of acute viral infection and chronic inflammation caused by underlying diseases or immunosuppressive therapy. These factors may jointly constitute the pathological and physiological basis for poor prognosis. Therefore, clinicians’ treatment strategies should adopt a more holistic perspective. While actively controlling CNS infections, it is necessary to strengthen nutritional support, carefully manage systemic inflammatory responses, and remain alert to the occurrence of complications to improve the overall prognosis of patients.

This study also found differences in prognostic factors among patients with different immune states. For immunocompetent patients, the occurrence of encephalitis and lower CD4+ T-cell counts were independent risk factors and important predictors of poor prognosis. This indicates that even in patients with immune function, the occurrence of encephalitis suggests more severe neurological damage, and immediate cell immune response ability has a protective effect on controlling infection and improving prognosis. In contrast, for immunocompromised patients, advanced age was another independent risk factor in addition to delayed treatment. This may be related to the fact that elderly patients have more comorbidities and decreased organ functional reserve, making clinical recovery more difficult when combined with immunosuppression and infection. Therefore, more individualized and refined management should be provided for these patients.

This study also has some limitations. Firstly, as a single-center retrospective study, the sample size is relatively small, which may lead to selection bias. In the comparison of baseline characteristics of immunocompromised and immunocompetent HZ patients with VZV-associated CNS infection, the absolute standardized mean differences (SMD) of some parameters were between 0.1-0.2, indicating some imbalance in the data between the two groups. This is mainly because the study is a retrospective study that relies on existing databases, where patients are passively grouped and matching is difficult, making the control group an “imperfect reference group”. This is also a common flaw in many retrospective studies. Meanwhile, all patients included in this study had typical shingles, and the representativeness of patients with zoster sine herpete with concurrent meningitis/encephalitis was insufficient, Previous studies have found that zoster sine herpete often complicates with CNS infections (35, 36, 42). Secondly, the prognosis assessment was based solely on outcomes at discharge, lacking long-term follow-up data, and reliance on a single time point limits generalizability. Subsequent studies should avoid these limitations. Lastly, the definition of immunocompromised in this study encompasses various etiologies (e.g., hematological diseases, solid tumors, organ transplantation, and autoimmune diseases). Different etiologies may have significant heterogeneity in their impact on the risk and prognosis of VZV-associated CNS infection. In the future, larger samples and multi-center prospective studies are needed to further validate the results of this study.

## Conclusion

In summary, the risk factors and prognosis of CNS infection in HZ patients with different immune function vary. Immunocompromised patients have a higher probability of developing encephalitis and a worse prognosis. Regardless of the patient’s immune status, delayed initiation of antiviral treatment after the onset of neurological symptoms is the most critical and modifiable factor leading to poor prognosis. Therefore, clinicians should maintain high vigilance for patients suspected of having VZV-associated CNS infection and adhere to the principle of early diagnosis and treatment to maximize the improvement of prognosis.

## Data Availability

The raw data supporting the conclusions of this article will be made available by the authors, without undue reservation.
